# A Multi-Drug Concentration Gradient Mixing Chip: A Novel Platform for High-Throughput Drug Combination Screening

**DOI:** 10.3390/bios14050212

**Published:** 2024-04-23

**Authors:** Jiahao Fu, Yibo Feng, Yu Sun, Ruiya Yi, Jing Tian, Wei Zhao, Dan Sun, Ce Zhang

**Affiliations:** 1 State Key Laboratory of Photon-Technology in Western China Energy, Institute of Photonics and Photon-Technology, Northwest University, Xi’an 710127, China; 2 Key Laboratory of Resource Biology and Biotechnology in Western China, Ministry of Education, School of Medicine, Northwest University, Xi’an 710127, Chinayi-ruiya@outlook.com (R.Y.);; 3 Huaxin Microfish Biotechnology Co., Ltd., Taicang 215400, China; 4 Center for Automated and Innovative Drug Discovery, Northwest University, Xi’an 710127, China

**Keywords:** multi-drug, mixing chip, microfluidic, drug screening

## Abstract

Combinatorial drug therapy has emerged as a critically important strategy in medical research and patient treatment and involves the use of multiple drugs in concert to achieve a synergistic effect. This approach can enhance therapeutic efficacy while simultaneously mitigating adverse side effects. However, the process of identifying optimal drug combinations, including their compositions and dosages, is often a complex, costly, and time-intensive endeavor. To surmount these hurdles, we propose a novel microfluidic device capable of simultaneously generating multiple drug concentration gradients across an interlinked array of culture chambers. This innovative setup allows for the real-time monitoring of live cell responses. With minimal effort, researchers can now explore the concentration-dependent effects of single-agent and combination drug therapies. Taking neural stem cells (NSCs) as a case study, we examined the impacts of various growth factors—epithelial growth factor (EGF), platelet-derived growth factor (PDGF), and fibroblast growth factor (FGF)—on the differentiation of NSCs. Our findings indicate that an overdose of any single growth factor leads to an upsurge in the proportion of differentiated NSCs. Interestingly, the regulatory effects of these growth factors can be modulated by the introduction of additional growth factors, whether singly or in combination. Notably, a reduced concentration of these additional factors resulted in a decreased number of differentiated NSCs. Our results affirm that the successful application of this microfluidic device for the generation of multi-drug concentration gradients has substantial potential to revolutionize drug combination screening. This advancement promises to streamline the process and accelerate the discovery of effective therapeutic drug combinations.

## 1. Introduction

Combinatorial drug treatments hold significant promise for the advancement of personalized medicine and the management of complex diseases. This approach harnesses the potential of multiple drugs to work together, creating synergies that can lead to improved treatment outcomes [1,2,3]. However, screening drug combinations and determining their appropriate concentrations are time-consuming and costly, and can take over 10 years and cost billions of dollars. Therefore, the development of technologies, including high-throughput screening methods [4,5,6,7], computational modeling [8,9,10], and microfluidic devices [11,12,13], is in high demand.

High-throughput cell screening typically relies on costly and complex methods such as porous plates with automated liquid transfer or manual operations, limiting their feasibility in small research centers. In contrast, microfluidic technology offers low-cost, precise control over nano-volume liquids, high integration, and efficient and cost-effective drug screening with minimal sample consumption [14,15,16]. Besides their requirement of few biological samples and reagents [17,18,19], microfluidic systems offer advantages in drug screening by creating a controlled biological microenvironment and allowing for high-resolution, real-time monitoring [20,21,22]. The integration of Quake’s valves into the microfluidic system allows for the automated and parallel processing of a large number of samples [23,24,25]. In the context of combinatorial drug discovery, this might mean rapidly screening thousands of drug combinations to assess their effects on cells or biological targets [26,27,28].

To overcome the challenges inherent to combinatorial drug screening, we present a novel microfluidic device designed to automatically generate drug concentration gradients within an array of micro-sized chambers. This device features a symmetrical chip layout enhanced with both upper and lower microvalves at each inlet, enabling the creation of different concentration gradients for three distinct drugs. This configuration facilitates the mixing of drugs with varying doses, spanning high to low concentrations. Through the integrated control of microvalves via a bespoke Matlab program, we can orchestrate the delivery of various doses of EGF, PDGF, and FGF into the microenvironment surrounding neural stem cells (NSCs). Utilizing the expression levels of Hes-5 in individual cells as a biomarker [29,30], we uncover that introducing individual growth factors at elevated concentrations promotes the differentiation of NSCs. Conversely, when NSCs are exposed to high doses of multiple drugs, we observe a reduction in the number of differentiated cells, which suggests the promotion of stem cell self-renewal. In summary, the successful deployment of our microfluidic device for the creation of multi-drug concentration gradients represents a significant leap forward in the realm of drug combination screening. Its capacity to deliver efficient, cost-effective evaluations of drug interactions signals a major advancement in supporting the development of new pharmacological agents and paves the way for personalized treatment methodologies.

## 2. Materials and Methods

### 2.1. Fabrication of Microfluidic Chips

In this study, we prepare the control and flow layers of multi-layer polydimethylsiloxane (PDMS) chips using lithography and soft lithography techniques [31]. The PDMS chip was designed in AutoCAD and fabricated using standard UV lithography with SU-8 3025, SU-8 3075 (Microchem, Westborough, MA, USA), and AZ-50X (AZ Electronic Materials, Luxembourg) photoresists. AZ-50X is used to create liquid channels with curved profiles, which are integrated with the rectangular profile channels of SU-8 3025 and SU-8 3075 to form a fluidic layer. This layer is then aligned and bonded with the control layer, also fabricated from SU-8 3025. For the fabrication process, 50 g of PDMS (10:1 monomer to catalyst ratio) is poured over the fluidic layer mold, degassed in a vacuum oven for one hour, and then cured at 80 °C for two hours. Subsequently, a thin PDMS layer is spin-coated onto the control-layer silicon wafer at 2200 rpm and baked for 10 min. After plasma treating the fluid and control layers, the layers are aligned using the ‘cross’ marks on each to achieve precise multi-layer bonding. The assembled PDMS chip undergoes further plasma treatment along with a glass substrate, is bonded, and then is post-cured in an 80 °C oven for over 8 h.

### 2.2. Numerical Simulation

In this study, we used COMSOL Multiphysics® 5.3 for numerical simulation. Based on the chip’s actual dimensions, a two-dimensional schematic was generated using COMSOL software, illustrating the three channels sequentially linked to five culture chambers to delineate the simulation boundaries. Their properties are configured to confine liquid flow to within the channels without wall adhesion. The simulation utilizes a multi-physics coupling interface integrating laminar flow and mass transfer. Inlet flow parameters of 3 mm/s, 5 mm/s, and 10 mm/s are assumed, with an initial substance concentration of 1 mol/m3 for simulation purposes.

### 2.3. Chip Operation

The microfluidic chips subjected to channel pressurization testing were sterilized via ultraviolet irradiation. The control channels of the chips were connected to miniature pneumatic solenoid valves (Festo, Esslingen, Germany), which were controlled using a custom MATLAB program (MathWorks, Natick, MA, USA). The optimal closing pressure of the PDMS membrane valves was determined, with the typical pressure range of the chips being between 25 and 30 psi. Prior to the chip’s use, its channels were filled with PBS and degassed. Before cell culturing, the chamber was coated with fibronectin (0.25 mg/mL; Merck, Vienna, Austria), followed by a continuous rinsing of the chip with PBS or cell culture medium.

### 2.4. Cell Culture and Loading

NSCs are isolated and cultured from the day-16 embyro-murine forebrain of Sprague Dawley (SD) rats and Hes5-GFP/Dcx-RFP double transgenic mice, which are subjected to experimental and chip cultivation procedures according to the established protocol [32,33,34]. The external tube holding the cells and fresh culture medium is pressurized by air containing 5% CO_2_ and delivered to the fluidic culture chamber by the programmed open–close of the PDMS membrane on valves on the chip or the on–off of the solenoid valves connected to the tube.

### 2.5. Image Acquisition and Data Analysis

For image acquisition, a Nikon Ti2-ECLIPSE microscope, with an automated translational stage and a digital CMOS camera (ORCA-Flash 4.0, Hamamatsu, Japan), and microscope (XDS-5, Bingyu, China) were used. Image acquisition was controlled by the Nikon microscope’s software (NIS components) and a custom-written control program, respectively. Bright field and fluorescence images were captured and analyzed using a customized MATLAB program.

## 3. Results

### 3.1. Design and Characterization of Microfluidic Chips

**Figure 1:**
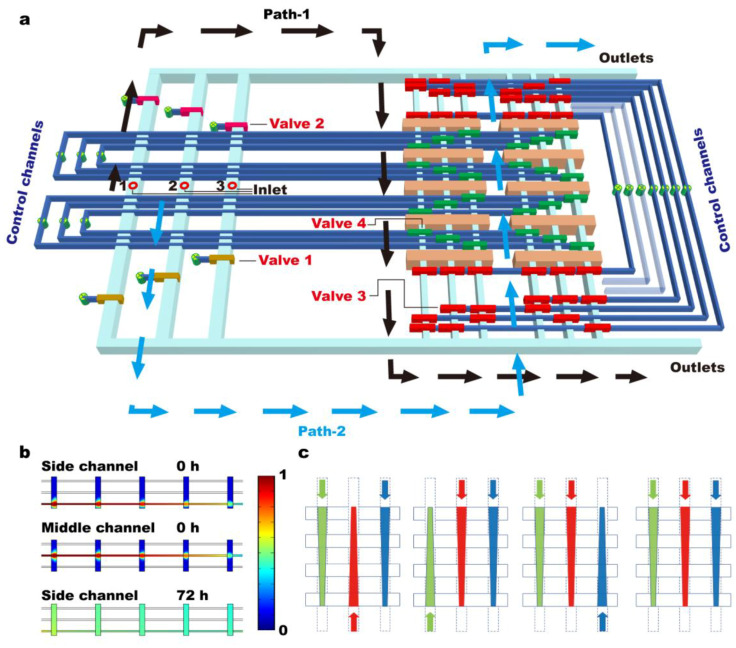
Design of the microfluidic device. (**a**) Three-dimensional configuration shows operational procedure of the microfluidic chip. Valves 1 (brown) and 2 (pink) function as the lower and upper inlet control valves, valve 3 (red) regulates the connection between microfluidic channels and culture chambers, and valve 4 (green) isolates the culture chambers. (**b**) Numerical simulation results indicate that when a fluid is directed through the array of culture chambers, it is gradually diluted. The direction of the concentration gradient depends on the paths, i.e., Path-1 for top-down and path-2 for the opposite direction. (**c**). Mixtures of different drug doses can be automatically generated by changing the direction of the concentration gradient, e.g., high + high + low.

To perform the high-throughput screening of combinatorial drugs, we propose a microfluidic chip which can generate mixtures of different drug doses with minimum effort (Figure 1). The primary material of these microfluidic chips, PDMS, is highly biocompatible, allowing for precise control of the microenvironment in long-term cell cultures and facilitating evaluations of drug screening and delivery [35,36,37,38,39,40,41,42]. The microfluidic chip is 6 cm in length, 5 cm in width, and 0.5 cm in height. It consists of multiple arrays of culture chambers (i.e., a grid of 2 × 5), which are interconnected by three microfluidic channels (Figure 1a). As shown In Figure 1a, Valves 1 and 2 function as the lower and upper inlet control valves, valve 3 (red) regulates the connection between the microfluidic channels and culture chambers, and valve 4 (green) isolates the culture chambers. To achieve precise control over the composition of combinatorial drug treatments (i.e., the direction, time and duration of drug input), all channels are independently controlled by Quake’s valves [31]. For example, by opening valves 1, 3, and 4, the drug from inlet-3 will be directed through Path-2, i.e., in the down-top direction through the array of culture chambers. Switching from valve 1 to valve 2 leads to changing the flow direction to top-down (Path-1).

Numerical simulation indicates that when drug inputs are directed through array of culture chambers, they are gradually diluted (Figure 1b and Figure S1). Paths, which liquid follows through the array of chambers, determine the direction of the concentration gradient; e.g., there is a high concentration in the top chambers when following Path-1. With a defined duration and input flow rate (i.e., 4 s and 5 mm/s), approximately 22.48% of the liquid is replaced in the top chamber, i.e., 0.2248 dilution. This concentration decreases to 0.2003, 0.1716, 0.1387, and 0.1128 of the input value in the subsequent chambers (Figure S2). Since the medium exchange takes place within an area of 250 µm by 790 µm, the liquid replacements in each one of the three microfluidic channels will hardly affect each other in a culture chamber of dimensions of 250 µm by 2000 µm (Figure 1b). Therefore, a combination of three drug doses can be generated by directing the flow via either Path-1 or Path-2. The resolution of the concentration gradient is determined by the number of chambers in the array, and the complexity of the combinatorial drugs is determined by the number of culture chamber arrays and inlets used.

**Figure 2:**
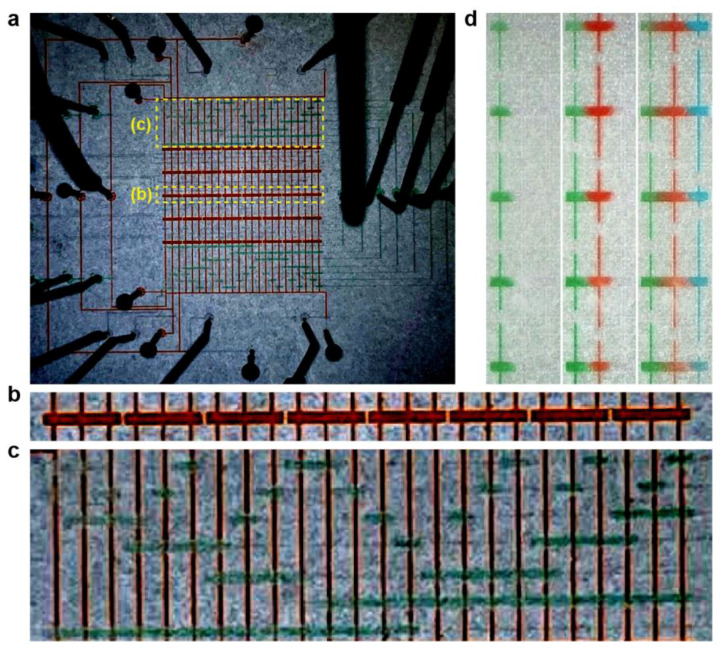
Operation of the microfluidic chip. (**a**) The microfluidic chip is produced, via soft lithography, with multi-layered structure, i.e., flow layers (red, such as (**b**)) and control layers (green, such as (**b**)). When pressurized by the connected tubes, the liquid in the chip can be manipulated with high accuracy. (**b**) The microfluidic chip has 8 culture chamber arrays, for which there are 5 culture chambers. These chambers are connected to 3 independently controllable microfluidic channels. (**c**) To control the liquids’ passage through each microfluidic channel, a three-control-one arrangement is employed. Using this approach, 7 thin Quake’s valves are needed to control 24 microfluidic channels. (**d**) Using green-, red-, and blue-colored food dyes, it is demonstrated that the concentration gradients of the three drugs can be generated independently.

To demonstrate the capacity of the proposed device, the microfluidic chip is fabricated using soft lithography and consists of two layers, i.e., the flow (red) and control layers (green) (Figure 2a and Figure S3). Its 40 culture chambers form a grid of 8 columns × 5 rows (Figure 2b). Since each column has three independent microchannels, it requires 24 independently controllable liquid inputs to achieve the claimed screening capacity. As is shown in Figure 2c, a minimum of seven valves (through the combination calculation of $C_{7}^{3}$) are necessary to control the 24 liquid inlet channels (Figure S1b). Consistent with our numerical simulation, inputs of green-, red-, and blue-colored food dyes do not disturb one another, i.e., show no signs of immature mixing at the initial stage (Figure 2d and Movie S1). By changing the direction of the concentration gradient over a period of time, the mixing of different color concentration gradients is achieved in the cavity (Figure S4 and Movie S2).

### 3.2. Overdose of Growth Factors Leads to NSCs’ Diminished Stemness

**Figure 3:**
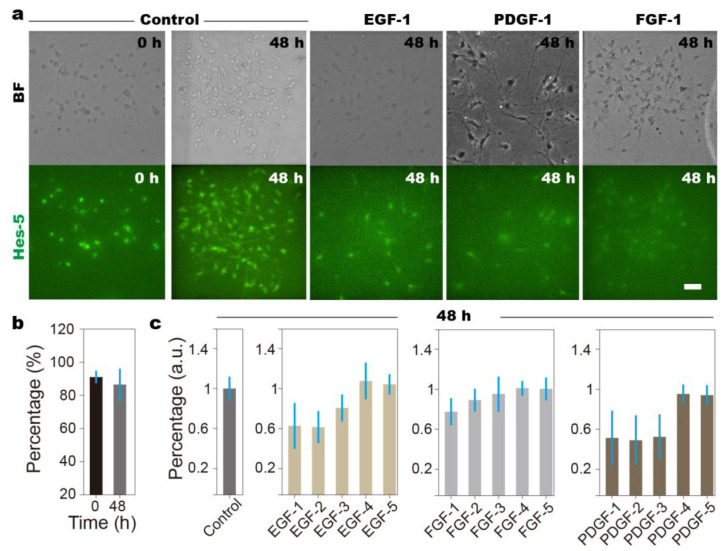
Live cell images reveal the regulatory effects of grow factor overdose on NSCs. (**a**) Bright field (BF) and fluorescent (Hes-5) images of NSCs when exposed to culture medium (control) and overdosed growth factors. EGF-1, -2, -3, -4, and -5 mean the high to low ends of the concentration gradient, i.e., 110 ng/mL, 100 ng/mL, 85 ng/mL, 70 ng/mL, and 55 ng/mL, respectively. FGF-1, -2, -3, -4, and -5 represent concentrations of 44 ng/mL, 40 ng/mL, 34 ng/mL, 28 ng/mL, and 22 ng/mL, respectively. PDGF-1, -2, -3, -4, and -5 represent concentrations of 220 ng/mL, 200 ng/mL, 170 ng/mL, 140 ng/mL, and 110 ng/mL, respectively. (**b**) By counting the cells losing Hes-5 fluorescence, the percentage of cells maintaining stemness can be estimated. The black and gray columns represent the results at 0 h and 48 h, respectively. Our results indicate that, with the culture medium, NSCs stemness can be well maintained. (**c**). At low growth factor concentrations, the number of differentiated NSCs is comparable to the control samples, while, at high growth factor concentrations, the percentage of differentiated NSCs reaches ~50%. Standard deviation is obtained from the average of at least 5 repeats. Scale bars denote 50 µm.

By directing cell-barring liquid through each column, NSCs are loaded into the culture chambers (Figure 3a). The number of Hes-5-positive cells is used as the indicator for NSCs’ stemness [43]. As a control, NSCs are maintained in culture medium during the 48 h experiments; the NSCs maintained in different culture chambers of the same array show no observable differences in cellular behavior and differentiation, indicating that the delivered nutrients meet the needs for cell survival (Figure 3b). To induce individual growth factors, we connect inlet-1 with a growth factor, and the other two inlets with culture medium (Figure 1a). With the addition of growth factors to the culture medium (1 μg/mL PDGF; 0.2 μg/mL FGF; 500 ng/ml EGF), concentration gradients are generated when the liquid is directed to pass through the array of culture chambers, which can be estimated to be ~110 ng/mL, 100 ng/mL, 85 ng/mL, 70 ng/mL, and 55 ng/mL for EGF-1, -2, -3, -4, and -5; ~44 ng/mL, 40 ng/mL, 34 ng/mL, 28 ng/mL, and 22 ng/mL for FGF-1, -2, -3, -4, and -5; and ~220 ng/mL, 200 ng/mL, 170 ng/mL, 140 ng/mL, and 110 ng/mL for PDGF-1, -2, -3, -4, and -5, respectively. It is observed that, at the highest doses, the addition of all growth factors leads to an increased number of differentiated NSCs, i.e., the cells losing Hes-5 fluorescence (Figure 1a), while their regulatory effects towards differentiation disappear at low growth factor concentrations (Figure 3c). For example, the number of Hes-5-positive cells exceeds even the control samples at EGF-4 and EGF-5, i.e., 70 ng/mL and 55 ng/mL. That is to say that individual growth factors act beneficially at low doses and produce contrary effects at high doses, i.e., a dose-dependent biphasic response, which is often observed in pharmacological experiments [44,45].

### 3.3. Combinatorial Treatment Reveals Logic Rules of Growth Factor Affecting NSCs Stemness

**Figure 4:**
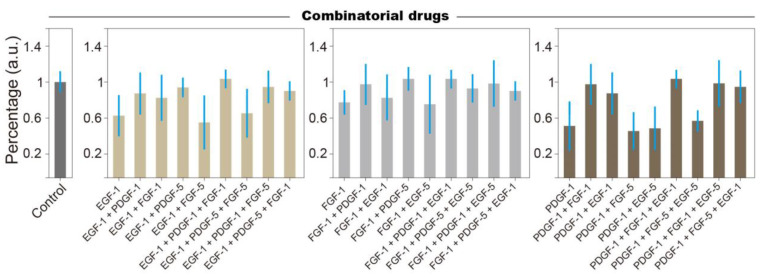
Regulatory effects of combinatorial drugs on NSCs’ stemness. Using the number of Hes-5-positive cells as the indicator, we investigate the effects of multiple high- and low-dose growth factors on NSCs. Standard deviation is obtained from the average of at least 5 repeats.

To introduce multiple growth factors into the culture chamber, two or three inlets are connected to the growth factors (Figure 1a). A combination of high and low doses are generated by directing liquid via Path-1 or Path-2 (Figure 1c), e.g., 1 + 5 + 5 represents high + low + low (Figure 4). The behavior of NSCs after being maintained on the chip under different drug conditions for 48 h reveals several rules: (1) the negative effects (i.e., differentiation) of a single growth factor overdose can be mediated by the overdosing another growth factors; (2) even though the addition of single growth factors at low concentrations helps maintain NSCs’ stemness, the addition of a low-concentration growth factor generates no observable effects on either control or differentiated NSCs except for PDGF-5; and (3) NSCs tend to be irresponsive under complex environmental conditions, i.e., multiple growth factors. Notably, the differentiation of NSCs induced by overdoses of EGF and FGF can be mediated by low concentrations of PDGF, while the differentiation of NSCs induced by a PDGF overdose cannot be interfered with by low doses of EGF and FGF. These results indicate that PDGF, as a promotor of either NSCs’ stemness or differentiation, has stronger effects compared to other growth factors [46]. We suspect that the signaling pathways activated by PDGF are digital, meaning that cascade reactions are triggered by trivial quantities of stimuli [47,48], while the signaling pathways of other growth factors are analog, the amplitude of which is concentration-dependent [49].

## 4. Discussion

Our research presents a groundbreaking microfluidic device capable of efficiently generating multi-drug concentration gradients, revolutionizing the screening process for combinatorial drug therapies. The device’s innovative design allows for the simultaneous creation of multiple drug gradients and the real-time monitoring of cellular responses, particularly NSCs. By employing this device, we have uncovered the dose-dependent biphasic responses to growth factors in the differentiation of NSCs. Our findings demonstrate that high concentrations of individual growth factors promote differentiation, whereas lower concentrations maintain NSC stemness. Moreover, the introduction of multiple growth factors reveals complex interaction patterns that modulate NSC outcomes, highlighting the importance of precise drug dosing in combinatorial therapies. The device’s ability to screen thousands of drug combinations rapidly and cost-effectively holds immense potential for personalized medicine, enabling the discovery of optimal therapeutic drug combinations tailored to individual patient profiles. The integration of microfluidic technology into drug discovery streamlines the process, reduces the resources required, and accelerates the identification of effective drug synergies.

In summary, our multi-drug concentration gradient mixing chip offers a novel and powerful platform for high-throughput drug combination screening. It has the potential to significantly advance our understanding of drug interactions and their effects on cellular behavior, paving the way for the development of more effective, personalized treatment strategies for complex diseases.

## Data Availability

Data underlying the results presented in this paper are not publicly available at this time but may be obtained from the authors upon reasonable request.

## Supplementary Materials

The following supporting information can be downloaded at https://www.mdpi.com/article/10.3390/bios14050212/s1, Figure S1: COMSOL simulation showing the dynamic process of drug diffusion in the array of culture chambers; Figure S2: Estimation of the concentration gradient generated in the array of cultivation chambers using numerical simulation and fluorescence intensity analysis; Figure S3: Design of the microfluidic chip using AutoCAD software; Figure S4: Comparison of results after gradient, mixing and diffusion of red, green, and blue pigments in different directions. Movie S1: Green, red, and blue food dyes are introduced into the cell culture chamber; Movie S2: Over time, the intermixing of concentration gradients of different colors (green, red, and blue) is achieved within the cavity.
