# Supplementary material for: A Multi-Drug Concentration Gradient Mixing Chip: A Novel Platform for High-Throughput Drug Combination Screening
Source: Biosensors (Basel). 2024 Apr 23;14(5):212. doi: 10.3390/bios14050212 (PMC11117479; doi:10.3390/bios14050212)
Supplement: Supplementary file 1 [file biosensors-14-00212-s001.zip › Supplementary information.pdf]

Supplementary Information

Multi-drug Concentration Gradient Mixing Chip: A Novel Platform for High-throughput Drug Combination Screening

Jiahao Fu [1, &], Yibo Feng [1, &], Yu Sun [2], Ruiya Yi [2], Jing Tian [2,3,4], Wei Zhao [1], Dan Sun [1,3,4*], and Ce Zhang [1,3*]

[1] State Key Laboratory of Photon-Technology in Western China Energy, Institute of Photonics and Photon-Technology, Northwest University, Xi'an, 710127, China

[2] Key laboratory of Resource Biology and Biotechnology in Western China, Ministry of Education, School of Medicine, Northwest University, Xi'an 710127, China.

[3] Huaxin Microfish Biotechnology Co., Ltd, Taicang, 215400, China

[4] Center for Automated and Innovative Drug Discovery, Northwest University, Xi'an, 710127, China

& These authors contribute equally to this work.

* Correspondence: zhangce.univ@gmail.com (C.Z), sund@nwu.edu.cn (D.S),

This PDF file includes:

Figure S1 to S4

Other supplementary materials for this manuscript include the following:
Movie S1 to S2

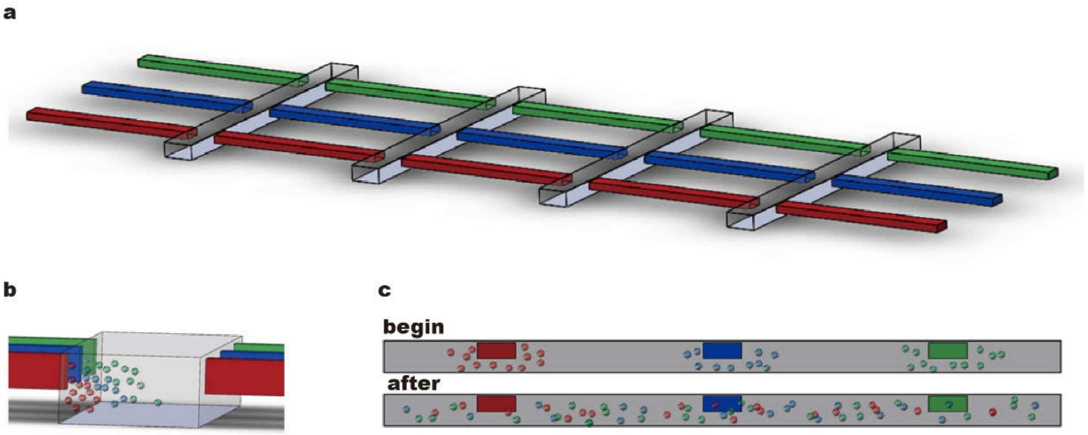

Figure S1: Comsol simulation showing the dynamic process of drug diffusion in the array of culture chambers. (a) Use green, red, and blue colors to represent the diffusion of three drugs through the array of culture chambers. (b) The diffusion of three drugs in the culture chamber. (c) Drug molecules contained within the culture chamber will gradually disperse over time.

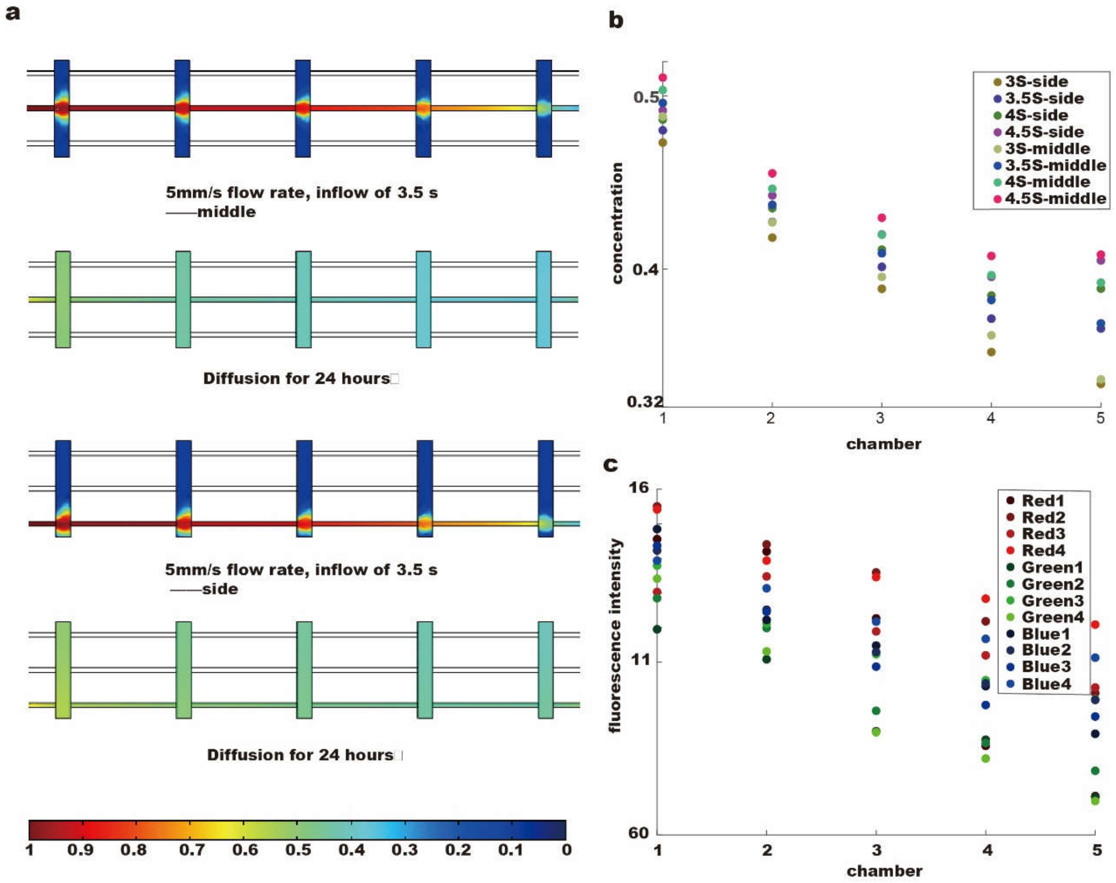

Figure S2: Estimation of the concentration gradient generated in the array of cultivation chambers using numerical simulation and fluorescence intensity analysis. (a) Numerical simulation shows the dynamic process of drug concentration gradient generation and reaching equilibrium after 24 hours. (b) Estimated concentration of drug molecules at different positions within the culture chamber and at different timepoints. (c) Measured fluorescent intensities at different position and timepoints, i.e., Red fluorescent dye (Rhodamine), green fluorescent dye (fluorescein isothiocyanate) and blue fluorescent dye (DAPI solution).

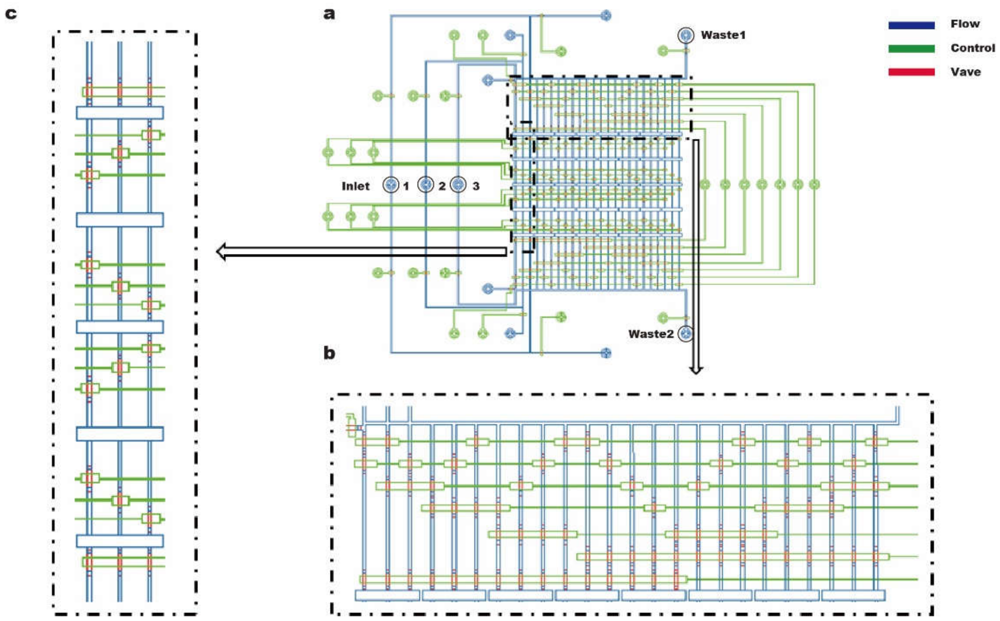

Figure S3: Design of the microfluidic chip using AutoCAD software. (a) The microfluidic chip comprises 3 inlets, 2 outlets, and 40 culture chambers. (b) Utilization of 7 valves calculated through the combination formula C_7^3 to regulate 24 inlet channels. (c) Control of liquid input to the culture chambers.

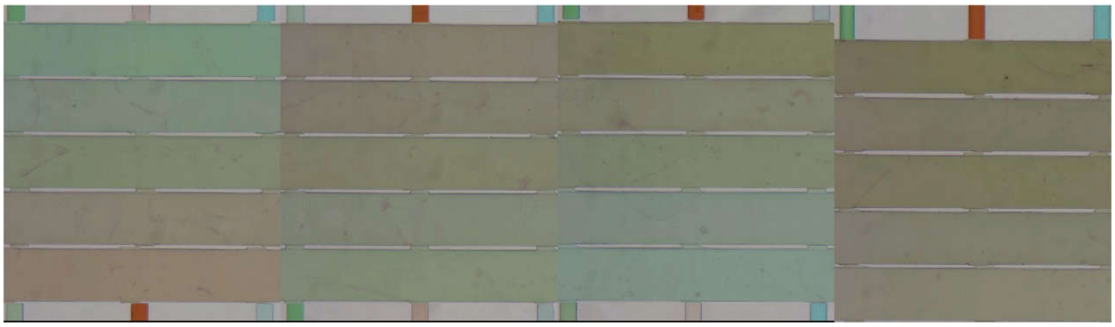

Figure S4: Comparison of results after gradient, mixing and diffusion of red, green, and blue pigments in different directions.

Movie S1. Green, red, and blue food dyes are introduced into the cell culture chamber.
Movie S2. Over time, the intermixing of concentration gradients of different colors (green, red, and blue) is achieved within the cavity.
