# Supplementary figures and images for: A Multi-Drug Concentration Gradient Mixing Chip: A Novel Platform for High-Throughput Drug Combination Screening
Source: Biosensors (Basel). 2024 Apr 23;14(5):212. doi: 10.3390/bios14050212 (PMC11117479; doi:10.3390/bios14050212)

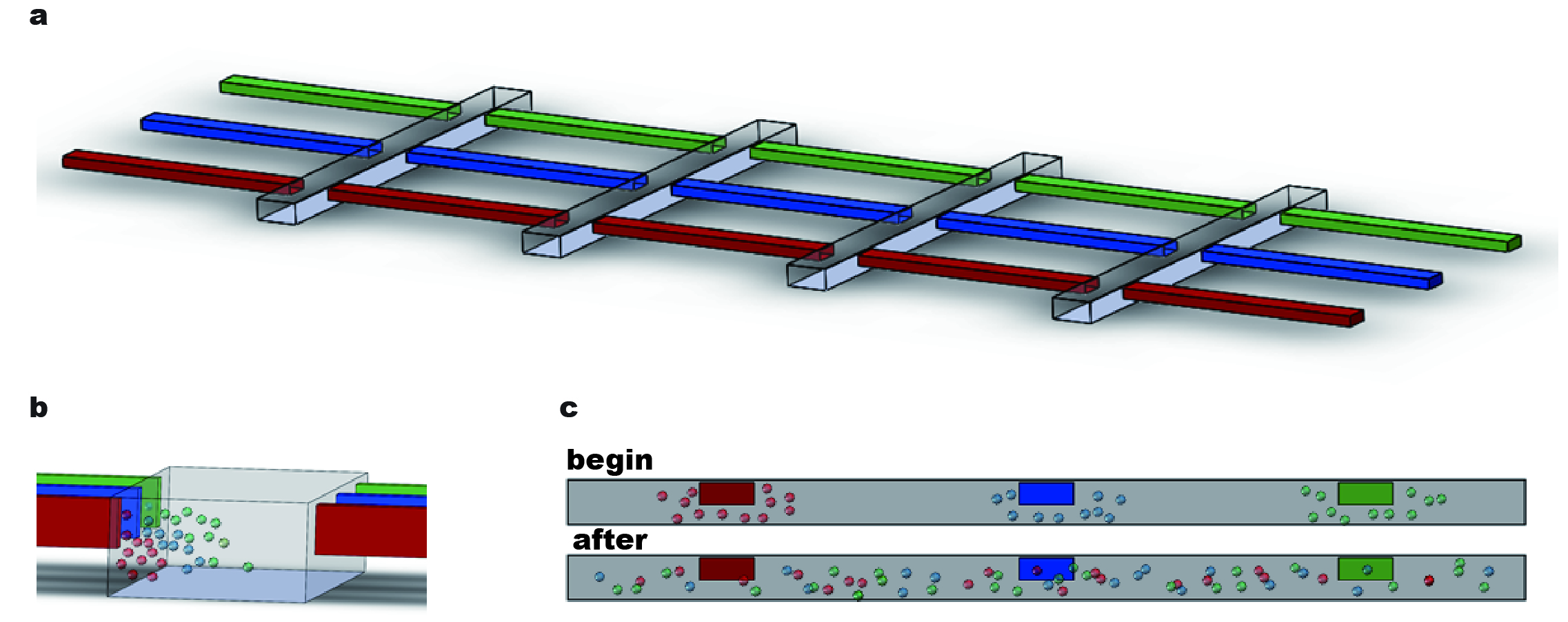

Supplement: Supplementary file 1 [file biosensors-14-00212-s001.zip › Figure S1.jpg]

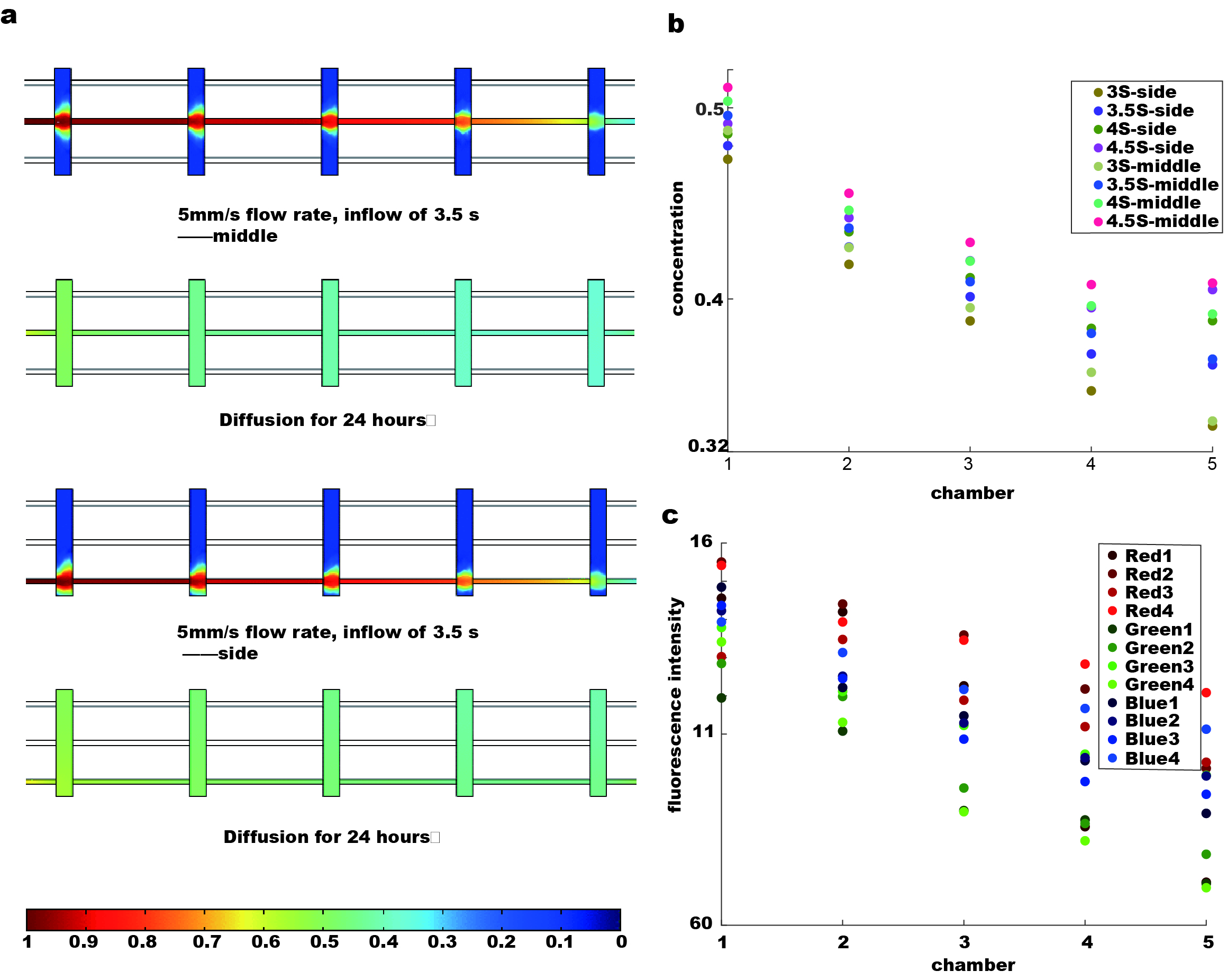

Supplement: Supplementary file 1 [file biosensors-14-00212-s001.zip › Figure S2.jpg]

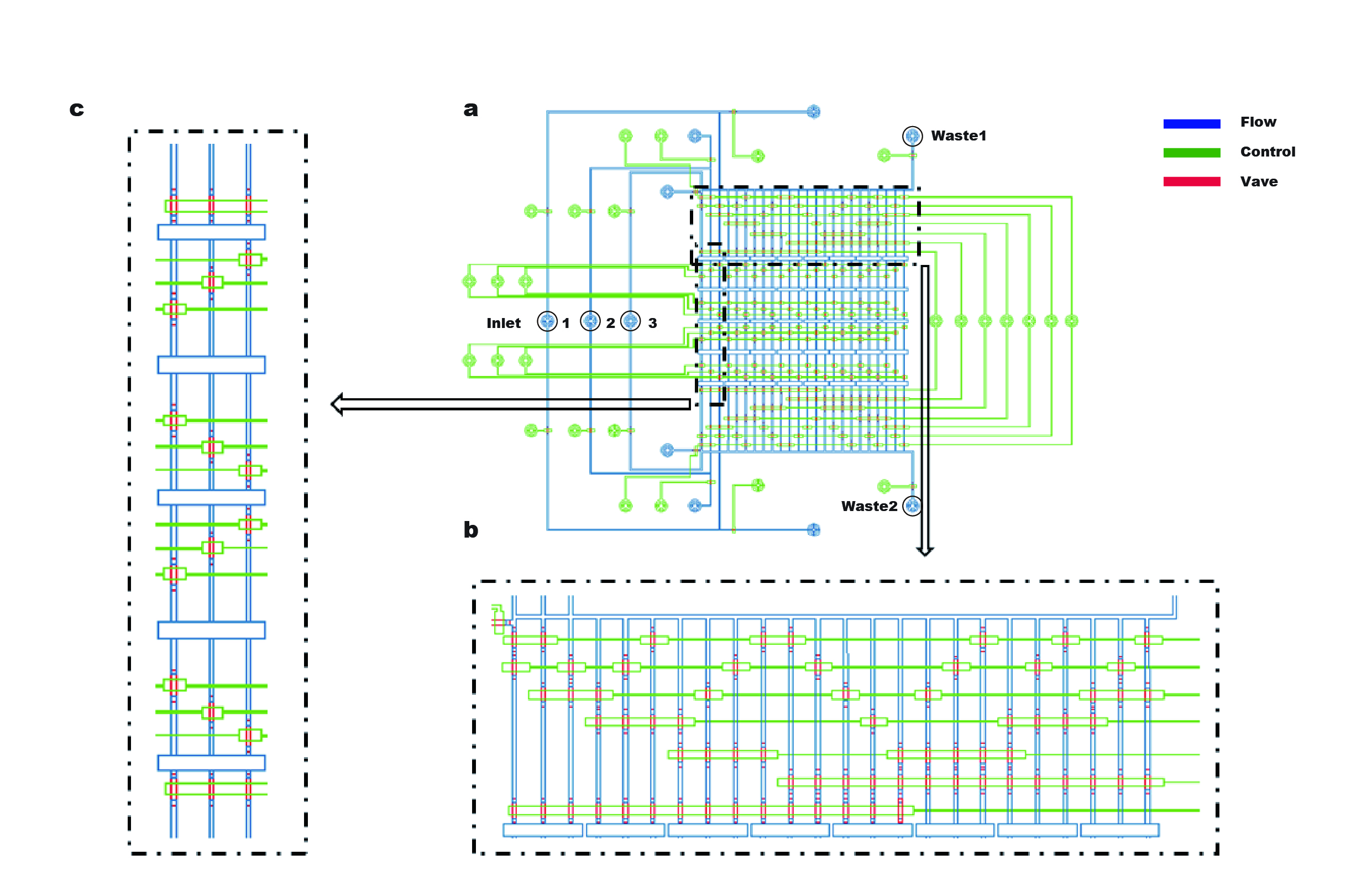

Supplement: Supplementary file 1 [file biosensors-14-00212-s001.zip › Figure S3.jpg]

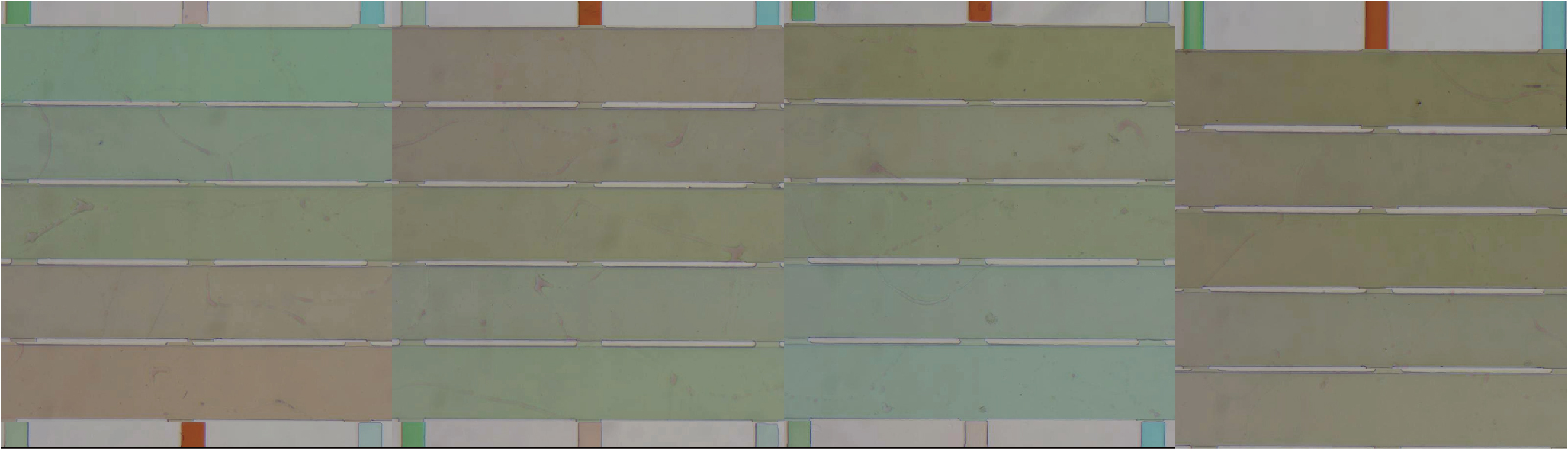

Supplement: Supplementary file 1 [file biosensors-14-00212-s001.zip › Figure S4.jpg]
